# Analysis of the substrate inhibition of complete and partial types

**DOI:** 10.1186/s40064-015-1082-8

**Published:** 2015-06-24

**Authors:** Masataka Yoshino, Keiko Murakami

**Affiliations:** Department of Biochemistry, Aichi Medical University School of Medicine, Yazako-Karimata 1-1, Nagakute, Aichi 480-1195 Japan

**Keywords:** Substrate inhibition, Partial inhibition, Complete inhibition, Quotient velocity plot, Phosphofructokinase

## Abstract

A simple graphical method was described for determining the kinetic parameters of substrate inhibition of complete and partial types. The method consists of plotting experimental data as $v/\left( {V_{max} - v} \right)$ versus the reciprocals of the substrate concentrations, where *V*_*max*_ represents the maximal velocity. The reaction rate constant of enzyme–substrate–inhibitor complex $(k^{\prime } /k)$ can be calculated from the ordinate intercept of the linear relationship between $v/\left( {V_{max} - v} \right)$ and the reciprocal of the substrate concentrations at the higher and inhibitory concentrations of the substrate: partial type $(k^{\prime } /k < 1)$ of the substrate inhibition gives straight lines intersecting with the ordinate at $(k^{\prime } /k)/( 1- k^{\prime } /k)$, whereas complete substrate inhibition $(k^{\prime } = 0)$ yields straight lines converging on the origin. The $K_{i}^{\prime }$ value also can be calculated from the slope by using the $k^{\prime } /k$ value determined. Validity of the method was confirmed by analyzing the substrate inhibition of phosphofructokinase II from *E. coli*. The present method provides a simple way for determining kinetic parameters of the substrate inhibition irrespective of complete and partial types.

## Background

Enzyme inhibition by its substrate in excess, substrate inhibition, is one of the common deviations from Michaelis–Menten kinetics, and means that the velocity curve of a reaction rises to a maximum as substrate concentration increases and then descends either to zero (complete inhibition) or to a non-zero asymptote (partial inhibition). Substrate inhibition is an extremely widespread phenomenon in enzyme kinetics, and plays critical regulatory roles in a number of metabolic pathways (Kaiser 1980; Kühl 1994; Reed et al. 2010). The simplest explanation of substrate inhibition implies the binding of two substrate molecules to the enzyme at the active sites and the non-catalytic inhibitory sites. Thus, determination of kinetic parameters including the dissociation constants of the substrates for the catalytic sites and inhibitory sites, and the reaction rate constants of the enzyme–substrate and enzyme–substrate–inhibitor complexes is necessary for the study on substrate inhibition. However, various kinetic methods for analyzing inhibition mechanism (Segel 1973; Cornish-Bowden 1974a, b; Eisenthal and Cornish-Bowden 1974; Baici 1981; Yoshino 1987) cannot be applied to substrate inhibition. In this report we describe a new graphical method for direct determination of the inhibition constants and the reaction rate constant of substrate inhibition. The present method consists of plotting experimental data as $v/(V_{max} - v)$ versus the reciprocal of the inhibitory substrate concentrations, and is applicable for analyzing partial and complete types of substrate inhibition. Validity of the method was confirmed by the analysis of the substrate inhibition of *E. coli* phosphofructokinase II.

## Theory and model

We now derive an equation for substrate inhibition. The enzyme, *E*, has two binding sites for its substrate *S*, a catalytic site for binding that can produce the product, *P*, and a non-catalytic or allosteric site that generates the product at a reduced rate. We may write the following reaction scheme:1

**Figure 1:**
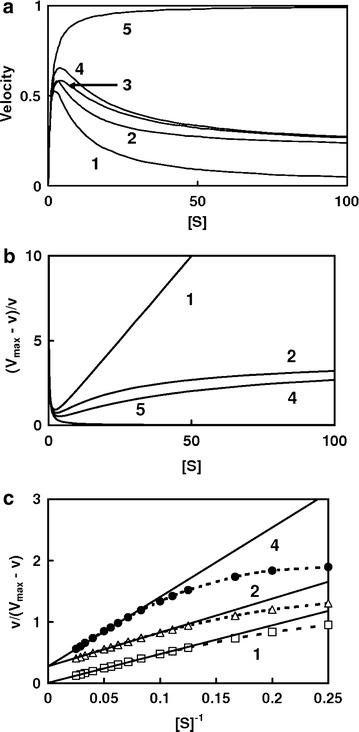
Substrate inhibition. Reaction for substrate inhibition was according to the Eq. (1). **a** Substrate saturation curves. Lines were drawn in accordance with Eq. (2). The following values of kinetic parameters were used for calculation. Rate constant, *V*_*max*_ = 1; *K*_*m*_ = 1. *Curve 1* complete substrate inhibition; *k* = 1, *k′* = 0, *K*_*m*_ = 1, $K_{{_{i} }}^{\prime } = 5$, *K*_*i*_ = ∞. *Curve 2* partial substrate inhibition; *k* = 1, *k′* = 0.2, *K*_*m*_ = 1, $K_{{_{i} }}^{\prime } = 5$, *K*_*i*_ = ∞. *Curve 3* partial substrate inhibition; *k* = 1, *k′* = 0.2, *K*_*m*_ = 1, $K_{{_{i} }}^{\prime } = 10$, *K*_*i*_ = 5. *Curve 4* partial substrate inhibition; *k* = 1, *k′* = 0.2, *K*_*m*_ = 1, $K_{{_{i} }}^{\prime } = 10$. *Curve 5* no substrate inhibition; *k* = 1, *k′* = 0, *K*_*m*_ = 1, $K_{{_{i} }}^{\prime } = \infty$, *K*_*i*_ = ∞. **b** Quotient velocity plot. (*V*_*max*_ − *v*)/*v* was plotted against the substrate concentrations. **c** Reciprocal of the quotient velocity plot. *Dotted lines* were theoretical curves, and *solid linear lines* were drawn within higher substrate concentration range.

We denote by *ES*_*1*_ and *ES*_*2*_, the substrates bound to the catalytic site and to the non-catalytic site, respectively, and further by *ES*_*1*_*S*_*2*_ two substrate molecules bound to both the catalytic and the non-catalytic sites. It is formally convenient to regard $K_{s} ,K_{{_{s} }}^{\prime } , \, K_{si} \;{\text{and}}\;K_{{_{si} }}^{\prime }$ as the dissociation constants of the enzyme–ligand complexes as described in Eq. (1): $K_{S} = k_{ - 1} /k_{ + 1} ,\;K_{S}^{\prime } = k_{ - 1}^{\prime } /k_{ + 1}^{\prime }$. A relationship among four dissociation constants of the enzyme–ligand complexes is $K_{si}^{\prime } /K_{si} = K_{{_{s} }}^{\prime } /K_{s}$. The substrate further affects the *V*_*max*_, that is to say the *ES*_*1*_*S*_*2*_ complex is assumed to break down at a different velocity from the *ES*_*1*_ complex, and the rate constants *k* and *k′* apply to the breakdown of the *ES*_*1*_ complex and *ES*_*1*_*S*_*2*_ complex, respectively. A rate equation for the reaction in Eq. (1) is derived according to the rapid equilibrium method. This system is based on the assumption that the overall rate of the reaction is limited by the breakdown of the ES complex to form free enzyme and product. This assumption has been widely accepted for most enzymes. Under the conditions where *k* is very small compared to $k_{ - 1}$, $K_{m} \approx k_{ - 1} /k_{ + 1} \approx K_{s}$; that is, *K*_*m*_ is essentially the dissociation constant of the ES complex. Thus;

2 $${v}{} = \frac{{V_{\hbox{max} } \cdot (1 + [S]/K_{Si}^{\prime } \cdot k^{\prime } /k) \cdot [S]}}{{[S](1 + [S]/K_{Si}^{\prime } ) + K_{m} (1 + [S]/K_{Si} )}}$$

where [*S*] is the concentration of the substrate. Figure 1 shows the substrate saturation curves under the various conditions of $K_{m} , \, K_{{_{i} }}^{\prime } , \, K_{i} \;{\text{and}}\;k^{\prime } /k$ values.

When $K_{Si} = \infty$ in Eq. (1), second substrate molecule can bind to only the enzyme–substrate complex, *ES*_*1*_, to an inactive or less active complex, *ES*_*1*_*S*_*2*_. This scheme is analogous to that for uncompetitive inhibition, and is the simplest model for substrate inhibition, which is widely accepted by most researchers (Dixon and Webb 1958; Cornish-Bowden 1974a, b; Reed et al. 2010). Equation (2) can be rearranged if $K_{Si} = \infty$.

3 $$\frac{v}{{V_{\hbox{max} } }} = \frac{{(1 + [S]/K_{Si}^{\prime } \cdot k^{\prime } /k) \cdot [S]}}{{(1 + [S]/K_{Si}^{\prime } ) \cdot [S] + K_{m} }}$$

General Eq. (2) is represented in the reciprocal form, and is rearranged as the form of “Quotient velocity plot” (Yoshino and Murakami 2009):

$$\frac{{V_{\hbox{max} } - v}}{v} = \frac{{[S]^{2} /K_{Si}^{\prime } \cdot (1 - k^{'} /k) + K_{m} (1 + [S]/K_{Si} )}}{{(1 + [S]/K_{Si}^{\prime } \cdot k^{\prime } /k) \cdot [S]}}$$

4 $$\frac{{V_{\hbox{max} } - v}}{v} = \frac{{[S]/K_{Si}^{\prime } \cdot (1 - k^{\prime } /k)}}{{(1 + [S]/K_{Si}^{\prime } \cdot k^{\prime } /k)}} + \frac{{K_{m} (1 + [S]/K_{Si} )}}{{(1 + [S]/K_{Si}^{\prime } \cdot k^{\prime } /k) \cdot [S]}}$$

The second term of Eq. (4) is negligible in the presence of higher and inhibitory concentrations of substrates. Thus, (*V*_*max*_ *−* *v*)/*v* can be approximated as follows:

$$\frac{{V_{\hbox{max} } - v}}{v} \approx \frac{{[S]/K_{Si}^{\prime } \cdot (1 - k^{\prime } /k)}}{{1 + [S]/K_{Si}^{\prime } \cdot k^{\prime } /k}}$$

Then,

5 $$\frac{{V_{\hbox{max} } - v}}{v} \approx \frac{{[S](1 - k^{\prime } /k)}}{{K_{Si}^{\prime } + [S] \cdot k^{\prime } /k}}$$

The relationship between (*V*_*max*_ *−* *v*)/*v* and the substrate concentration gives a hyperbolic curve under the conditions of higher substrate concentrations (Figure 1b, curves 2 and 4), and the double reciprocal form of Eq. (5) can be rearranged as follows:

6 $$\frac{v}{{V_{\hbox{max} } - v}} \approx \frac{{K_{Si}^{\prime } }}{{1 - k^{\prime } /k}} \cdot \frac{1}{[S]} + \frac{{k^{\prime } /k}}{{1 - k^{\prime } /k}}$$

Equation (6) shows that $v/\left( {V_{max} {-}v} \right)$ is a linear function of the reciprocal of the substrate concentrations: the straight lines intersect with the ordinate at $(k^{\prime } /k)/(1 - k^{\prime } /k)$ (Figure 1c, lines 2 and 4).

In complete inhibition with *k′* = 0, Eq. (5) can be reduced as follows:

7 $$\frac{{V_{\hbox{max} } - v}}{v} \approx \frac{[S]}{{K_{Si}^{\prime } }}$$

The relationship between (*V*_*max*_ *−* *v*)/*v* and [S] of complete inhibition gives straight lines under the higher concentrations of substrate (Figure 1b, curve 1). Complete type of the substrate inhibition shown in Eq. (7) can be arranged as,

8 $$\frac{v}{{V_{\hbox{max} } - v}} \approx \frac{{K_{i}^{\prime } }}{[S]}$$

Thus, Eq. (8) is a linear function of the reciprocal of the substrate concentration converging on the origin with the slope of $K_{{_{Si} }}^{\prime }$ (Figure 1c, line 1).

We can calculate the $k^{\prime } /k\;{\text{and}}\;K_{{_{Si} }}^{\prime }$ values for the substrate inhibition of complete and partial types as follows:

1. The $k^{\prime } /k$ value is calculated from the y-intercept
2. The $K_{{_{Si} }}^{\prime }$ value can be calculated from the slope of the lines by using $k^{\prime } /k$ values determined.

## Application to the practical case

Phosphofructokinase is a key enzyme in glycolysis, and is controlled by various allosteric effectors (Ramaiah 1974). Of regulatory ligands, ATP the substrate also acts as an inhibitor of the enzyme: inhibition of phosphofructokinase by ATP is the representative of the substrate inhibition, and its biological significance was discussed in relation to the glycolytic control (Reed et al. 2010). Here we attempt to analyze the inhibition of *E. coli* phosphofructokinase II (encoded by *pfk*B) by ATP with the present method.

**Figure 2:**
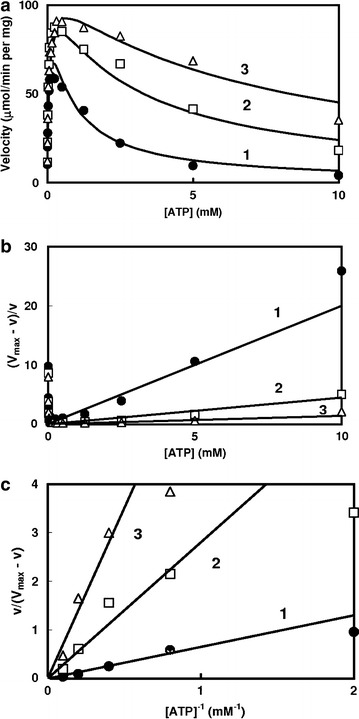
Substrate inhibition of phosphofructokinase II (encoded by *pfk*B) from *E. coli*. **a** Substrate saturation curve. Phosphofructokinase activity was determined by following the decrease in the NADH concentration using aldolase, triose phosphate isomerase and glycerol 3-phosphate dehydrogenase as coupling enzymes. The reaction was started by the addition of phosphofructokinase, and the decrease in absorbance at 340 nm was recorded. **a** Substrate saturation curves. Points were experimental data and lines were theoretically drawn from Eq. (1), using the following parameter values: *V*_*max*_ = 110 μmol/min per mg. *K*_*m*_ = 0.065 mM. *Curve 1* $K_{i}^{\prime }$ = 0.65 mM. *Curve 2* $K_{i}^{\prime }$ = 2.8 mM. *Curve 3* $K_{i}^{\prime }$ = 7 mM. **b** Quotient velocity plot. **c** Reciprocal of the quotient velocity plot. $K_{i}^{\prime }$ values were calculated from the slope of the lines at the higher substrate concentration.

*E. coli* phosphofructokinase II showed non-allosteric properties, and was subject to typical substrate inhibition with respect to ATP (Figure 2a). *V*_*max*_ value was calculated from the double reciprocal plot under the conditions of lower substrate concentrations (data not shown). $\left( {V_{max} - v} \right)/v\;{\text{and}}\;v/\left( {V_{max} - v} \right)$ were plotted against ATP concentrations and the reciprocal of ATP at higher concentration range, respectively (Figure 2b, c). Relationship between *v/*(*V*_*max*_ − *v*) and the reciprocal of ATP concentrations gave straight lines converging on the origin, indicating the inhibition to be of complete type. We calculated the apparent $K_{{_{Si} }}^{\prime }$ values to 0.65, 2.8 and 7 mM in the presence of 0.1, 0.5 and 5 mM fructose 6-phosphate, respectively from the slope of the line in Figure 2c.

## Discussion

Substrate inhibition is explained by the binding of two substrate molecules at the active sites and the inhibitory sites. Enzyme-substrate complex, *ES*_1_ can generate a reaction product; however, *ES*_1_*S*_2_ complex, the enzyme that binds two substrate molecules at the active site and the inhibitory site, breaks down at a reduced velocity than *ES*_1_ complex. This inhibition is classified as being of partial type (*k′/k* < *1*). On the other hand, when *ES*_1_*S*_2_ complex cannot produce the product, that is, *k′* = 0, the inhibition is called complete substrate inhibition.

The purpose of the study on enzyme inhibition is determination of inhibition constants and inhibition types. Several graphical methods have been used for analyzing kinetic parameters (Dixon 1953; Cornish-Bowden 1974a, b; Eisenthal and Cornish-Bowden 1974; Baici 1981; Yoshino 1987; Yoshino and Murakami 2009); however, these methods cannot be applied for the analysis of substrate inhibition. Analysis of the substrate inhibition was reported on fructose 1,6-bisphosphatase (Vargas et al. 1990), but universal analyzing methods for substrate inhibition, in particular, partial substrate inhibition have not been presented to date. The present work describes a new graphical method for analyzing substrate inhibition: the method consists of plotting experimental data as *v/*(*V*_*max*_ − *v*) versus 1/[S] at higher and inhibitory concentrations of the substrate: this plot can decide whether the substrate inhibition is of the complete or the partial type, and further can determine dissociation constant of the *ES*_1_*S*_2_ complex.

The present study serves as a useful graphical tool for determining substrate inhibition types and inhibition parameters such as $k^{\prime } /k$ and $K_{Si}^{\prime }$ values, although it requires *V*_*max*_ values for calculation. This plot can contribute to analysis of the inhibition mechanism and metabolic control. Recent kinetic studies use nonlinear regression analysis with computer simulation methods; thus, present plot and various linearization methods may be useful for initial orientation regarding the action of two substrate molecules.

## Conclusion

A simple graphical method for determining the inhibition constant of substrate inhibition was presented. Plot of *v*/(*V*_*max*_ − *v*) versus 1/[S] gave a linear relationship under the conditions of higher and inhibitory concentrations of substrate. The rate constant of the enzyme–substrate–inhibitor complex (*k′/k*) and $K_{Si}^{\prime }$ value were determined from the ordinate intercept and the slope, respectively. Inhibition of phosphofructokinase by the excess substrate ATP was analyzed.

## Methods

The source of materials used in this work as follows: NADH, glucose 6-phosphate, aldolase, triose phosphate isomerase, glycerol 3-phosphate dehydrogenase. Phosphofructokinase II (encoded by *pfkB*) was purified from the archived clone of the pfkII-overexpressed *E. coli* mutant, and the determination of the activity was described previously (Ogawa et al. 2007). Phosphofructokinase activity was determined by following the decrease in the NADH concentration using aldolase, triose phosphate isomerase and glycerol 3-phosphate dehydrogenase as coupling enzymes. Reaction mixture of 1 mL contained 0.1 M MOPS-KOH (pH 7.2), various concentrations of ATP, 5 mM MgC1_2_, 0.1 mM NADH, l mM dithiothreitol, l unit of aldolase, 3 units of triose phosphate isomerase, and 8 units of glycerol 3-phosphate dehydrogenase in the presence of 0.1, 0.5, and 5 mM fructose 6-phosphate. The reaction was started by the addition of phosphofructokinase, and the decrease in absorbance at 340 nm was recorded.
